# Algorithmic portfolio tilting to harvest higher moment gains

**DOI:** 10.1016/j.heliyon.2020.e03516

**Published:** 2020-03-05

**Authors:** Kris Boudt, Dries Cornilly, Frederiek Van Holle, Joeri Willems

**Affiliations:** a Universiteit Gent, Sint-Pietersplein 5, 9000 Gent, Belgium; b Vrije Universiteit Brussel, Pleinlaan 2, 1050 Brussel, Belgium; c Vrije Universiteit Amsterdam, De Boelelaan 1105, 1081 Amsterdam, Netherlands; d SYZ Asset Management, Rue du Commerce 3, 1204 Genève, Switzerland; e Degroof Petercam Asset Management, Guimardstraat 18, 1040 Brussel, Belgium

**Keywords:** Mean-variance-skewness-kurtosis, Non-normality, Portfolio allocation, Tilting, Statistics, Finance, Banking, Econometrics, Operations management, Business, Economics, Information science, Industry

## Abstract

Many financial portfolios are not mean-variance-skewness-kurtosis efficient. We recommend tilting these portfolios in a direction that increases their estimated mean and third central moment and decreases their variance and fourth central moment. The advantages of tilting come at the cost of deviation from the initial optimality criterion. In this paper, we show the usefulness of portfolio tilting applied to the equally-weighted, equal-risk-contribution and maximum diversification portfolios in a UCITS-compliant asset allocation setting.

## 0 Introduction

Many of the popular risk-based portfolios are optimized according to an optimality criterion that does not take into account higher order portfolio return moments (see e.g. Lee (2011)). Typical examples from the literature on risk-based asset allocation include the equally-weighted and equal-risk-contribution portfolios (Maillard et al. (2010)). A further example is the maximum diversification portfolio of Choueifaty and Coignard (2008), which maximizes the ratio between the weighted average standard deviation of the portfolio components and the portfolio standard deviation. These portfolios may still be efficient for investors with mean-variance preferences, as discussed in Ardia and Boudt (2015). They are, however, likely to be inefficient for investors whose preferences depend on the higher order moments of the portfolio return distribution.

Several solutions have been proposed in terms of portfolio allocation methods that take the higher order moments into account (see e.g. Jondeau et al. (2007) and Boudt et al. (2012)). In this paper, we study the problem of improving portfolios that are inefficient from a mean-variance-skewness-kurtosis (MVSK) perspective, where skewness and kurtosis are defined as the non-standardized third and fourth central portfolio moments. We do so by tilting such inefficient portfolios to an allocation that is more MVSK efficient, while keeping the good properties of the initial portfolio weights.^1^ The proposed methodology is inspired by the shortage minimization framework of Briec et al. (2007).

We expect MVSK portfolio tilting to be especially useful in asset allocation. We therefore illustrate the methodology by tilting three common risk-based asset allocation portfolios in a UCITS-compliant setting, where the investment universe consists of 12 broad asset class indices. We find that the tilted risk-based portfolios achieve superior out-of-sample moments while still showing the characteristics of the initial allocation criterion.

The remainder of the article is organized as follows. Section 1 introduces our notation. Section 2 presents the proposed methodology for portfolio tilting. Section 3 documents the usefulness of the method in a UCITS-compliant asset allocation framework. We end the paper with a conclusion. The supplementary appendix provides an example using the R package **mvskPortfolios**.

## 1 Notation

We consider the decision problem of optimizing a portfolio invested in *N* assets with weight vector $w\equiv {\left(w_{1},\ldots ,w_{N}\right)}^{\prime }$. The optimality of the portfolio weights is evaluated with respect to a reference portfolio $w_{0}$ and the first four portfolio return moments. For those portfolio return moments, accurate estimators for the corresponding comoment matrices have been proposed by Martellini and Ziemann (2010) and Boudt et al., 2020a, Boudt et al., 2020b, among others. The interpretation and reliable estimation of comoment matrices greater than four is a subject for further research. It is also an open research question whether the integration of portfolio moments greater than four in portfolio optimization improves the out-of-sample welfare of the investor (see e.g. Jondeau and Rockinger (2006)). Investors interested in portfolio optimization using all moments can directly optimize the portfolio return distribution, as in Haley and McGee (2006). This requires to re-estimate the portfolio return distribution for every candidate portfolio vector **w**. The computational advantage of our MVSK tilting portfolio is that the optimization does not require such re-estimation.

### 1.1 Asset returns, their comoments and the portfolio moments

The asset return over the investment horizon is denoted by $r\equiv {\left(r_{1},\ldots ,r_{N}\right)}^{\prime }$. Its mean, covariance, coskewness and cokurtosis matrices are

(1) $$\mu \equiv E\left[r\right],\Sigma \equiv E\left[(r-\mu ){\left(r-\mu \right)}^{\prime }\right],\Phi \equiv E\left[(r-\mu ){\left(r-\mu \right)}^{\prime }⊗{\left(r-\mu \right)}^{\prime }\right],\Psi \equiv E\left[(r-\mu ){\left(r-\mu \right)}^{\prime }⊗{\left(r-\mu \right)}^{\prime }⊗{\left(r-\mu \right)}^{\prime }\right],$$

where ⊗ stands for the Kronecker product. These comoments determine the portfolio higher order moments. Define $m_{q}\equiv E\left[{\left(w^{\prime }r-w^{\prime }\mu \right)}^{q}\right]$ as the *q*-th central portfolio moment, implicitly depending on the weights **w**. We have that $m_{2}\equiv w^{\prime }\Sigma w$, $m_{3}\equiv w^{\prime }\Phi (w⊗w)$ and $m_{4}\equiv w^{\prime }\Psi (w⊗w⊗w)$. We denote the portfolio tracking error volatility by the volatility of the difference in portfolio composition:

(2) $${\text{TE}}_{\text{vol}}(w)=\sqrt{{\left(w-w_{0}\right)}^{\prime }\Sigma (w-w_{0})}.$$

### 1.2 Optimality of the reference portfolio

We are agnostic about the construction of the reference portfolio $w_{0}$. It can be expert-based, algorithmic or a combination of both. The proposed MVSK tilting requires that the investor elicit an optimality criterion $h_{\text{ref}}(\cdot )$ for which $w_{0}$ is the optimal solution. This criterion is a function of the portfolio weights **w**, the asset return moments ***μ***, **Σ**, **Φ** and **Ψ** and possibly other parameters stacked into the vector ***ζ***:

(3) $$w_{0}\equiv {\mathrm{argmin}}_{w\in C}\;h_{\text{ref}}(w\;|\;\mu ,\Sigma ,\Phi ,\Psi ,\zeta ),$$

where C is the feasible set. It is always possible to find a criterion for which this reference portfolio is optimal. The simplest criterion is the tracking error volatility in (2), which is obviously minimized when $w=w_{0}$.

In the application, we consider three examples of $h_{\text{ref}}$-functions. The first equals the Herfindahl index of the portfolio weights:

(4) $$h_{\text{EW}}(w\;|\;\mu ,\Sigma ,\Phi ,\Psi ,\zeta )=\sum_{i=1}^{N}w_{i}^{2},$$

which is minimized by the equally-weighted portfolio. DeMiguel et al. (2009) refer to this portfolio as the 1/N allocation. The portfolio uses a naive approach to diversification as it ignores the heterogeneity in the component return distributions when optimizing the weights. The second objective function is the sum of squared deviations of the *N* percentage risk contributions with respect to 1/N:

(5) $$h_{\text{ERC}}(w\;|\;\mu ,\Sigma ,\Phi ,\Psi ,\zeta )=\sum_{i=1}^{N}{\left(\text{\%}RC_{i}-\frac{1}{N}\right)}^{2},$$

with $\text{\%}RC_{i}\equiv \frac{w_{i}{\left[\Sigma w\right]}_{i}}{w^{\prime }\Sigma w}$ the percentage volatility contribution of asset *i*. The solution to this optimization is the equal-risk contribution portfolio (Maillard et al. (2010)). The third objective that we consider is the negative of the diversification ratio (Choueifaty and Coignard (2008)):

(6) $$h_{\text{DR}}(w\;|\;\mu ,\Sigma ,\Phi ,\Psi ,\zeta )=-\frac{w^{\prime }\sqrt{\text{diag}(\Sigma )}}{\sqrt{w^{\prime }\Sigma w}},$$

where $\text{diag}(\Sigma )={\left(\Sigma_{11},\ldots ,\Sigma_{pp}\right)}^{\prime }$ is the N×1 vector containing the diagonal elements of **Σ**.

Note that the framework has a large scope and also includes the mean-variance efficient portfolios of Markowitz (1952) and the minimum modified Value-at-Risk and Expected Shortfall portfolios of Boudt et al., 2008, Boudt et al., 2012, among others. Moreover, the function $h_{\text{ref}}$ is not necessarily the one used to determine $w_{0}$. For example, it is possible to construct the maximum diversification portfolio with weights $w_{0}$ by minimizing $h_{\text{DR}}$, but use $h_{\text{ref}}={\text{TE}}_{\text{vol}}$ when tilting the portfolio.

## 2 MVSK portfolio tilting

We assume that the investor is willing to sacrifice a margin *κ* of the value of the reference objective function in order to improve the portfolio performance in terms of an increase in the mean and skewness and a decrease in the variance and the kurtosis. We impose these improvements through inequality constraints, as can be seen in Table 1, where the minimum levels of improvements in the mean, variance, skewness and kurtosis are $\delta_{\mu }$, $\delta_{\Sigma }$, $\delta_{\Phi }$, and $\delta_{\Psi }$, respectively.

**Table 1:** Impact of MVSK tilting on portfolio criteria.

Deterioration of reference objective	*h*_ref_(**w** | ***μ***,**Σ**,**Φ**,**Ψ**,***ζ***)≤*h*_ref_(**w**_0_ | ***μ***,**Σ**,**Φ**,**Ψ**,***ζ***)+*κ*
Increase in mean return	$w^{\prime }\mu \geq w_{0}^{\prime }\mu +\delta_{\mu }$
Decrease in variance	$w^{\prime }\Sigma w\leq w_{0}^{\prime }\Sigma w_{0}-\delta_{\Sigma }$
Increase in skewness	$w^{\prime }\Phi (w⊗w)\geq w_{0}^{\prime }\Phi (w_{0}⊗w_{0})+\delta_{\Phi }$
Decrease in kurtosis	$w^{\prime }\Psi (w⊗w⊗w)\leq w_{0}^{\prime }\Psi (w_{0}⊗w_{0}⊗w_{0})-\delta_{\Psi }$

Note: The parameters $\delta_{\mu }$, $\delta_{\Sigma }$, $\delta_{\Phi }$, $\delta_{\Psi }$ are positive when the MVSK tilted portfolio has preferential moments compared to the benchmark, at the cost of an increase of *κ* in $h_{\text{ref}}$.

Our approach to simultaneously look for improvements in expected return, variance, skewness and kurtosis is compatible with the accepted direction of preferences for the first four moments (see e.g. Scott and Horvath (1980)) and is closely related to the utility-based framework (Jondeau and Rockinger (2006), Harvey et al. (2010)). In the utility-based approach to non-Gaussian preferences, the expected utility is often approximated by a Taylor expansion, or more generally, a function

(7) $$U_{\alpha }(w)=\alpha_{1}w^{\prime }\mu -\alpha_{2}w^{\prime }\Sigma w+\alpha_{3}w^{\prime }\Phi (w⊗w)-\alpha_{4}w^{\prime }\Psi (w⊗w⊗w).$$

The vector ***α*** contains positive values and governs the relative importance of the higher moments with respect to the expected utility of the investor. Such an objective function is generally non-concave and hence it is impossible to guarantee a global maximum. In this regard, Briec et al. (2007) prove a primal-dual relationship between the primal, shortage-based approach followed in this paper and the dual approach of directly maximizing a non-concave expected utility approximation.

### 2.1 Choice of *δ* parameters

In the current formulation we have at least three unknowns: the tilted portfolio weights **w**, the minimum improvement levels $\delta_{\left(\cdot \right)}$ and the sacrifice parameter *κ*. In principle, we can assume that the investor will elicit these. In practice, however, this is not realistic and a data-driven calibration is required. To achieve this, we simplify the problem by assuming that the value of *κ* is fixed and that the $\delta_{\left(\cdot \right)}$'s are functions of a single *δ*.

The investor wishes to maximize this *δ*, thereby achieving more preferable portfolio moments at a maximum cost of *κ* in the reference objective function. The general MVSK portfolio tilting problem is then:

(8) $$\underset{\delta \in R,w\in C}{\text{maximize}}\;\delta \;\text{subject to}h_{\text{ref}}(w\;|\;\mu ,\Sigma ,\Phi ,\Psi ,\zeta )\leq h_{\text{ref}}(w_{0}\;|\;\mu ,\Sigma ,\Phi ,\Psi ,\zeta )+\kappa ,w^{\prime }\mu \geq w_{0}^{\prime }\mu +\delta_{\mu },w^{\prime }\Sigma w\leq w_{0}^{\prime }\Sigma w_{0}-\delta_{\Sigma },w^{\prime }\Phi (w⊗w)\geq w_{0}^{\prime }\Phi (w_{0}⊗w_{0})+\delta_{\Phi },w^{\prime }\Psi (w⊗w⊗w)\leq w_{0}^{\prime }\Psi (w_{0}⊗w_{0}⊗w_{0})-\delta_{\Psi },$$

where $\delta_{\mu }$, $\delta_{\Sigma }$, $\delta_{\Phi }$, and $\delta_{\Psi }$ are all increasing functions of *δ*. We denote this as ${\left(\delta_{\mu },\delta_{\Sigma },\delta_{\Phi },\delta_{\Psi }\right)}^{\prime }=g(\delta )$. The threshold *κ* determines the maximum deterioration in the reference function. In the extreme case of setting κ=∞, we have the MVSK optimization setup of Briec et al. (2007).

### 2.2 Choice of *κ* parameter

The optimization in (8) yields the optimal value of *δ* for a given value of *κ*, and increasing *κ* always increases the value of *δ*. To gauge this trade-off between low values of *κ* being more in line with the initial portfolio objective and higher values of *δ* yielding preferable moments, we recommend visualizing *δ* on a grid of *κ* values that ranges from zero to a pre-set upper bound $\bar{\kappa }$. Based on this, an investor or portfolio manager can select a value of *κ* that limits the decrease in the reference objective for a significant gain in moments. This choice will depend on the level, slope and curvature of the curve.

In the context of portfolio tilting, it is also natural to set *κ* at the highest possible value that respects an upper bound on the portfolio tracking error volatility ${\text{TE}}_{\text{vol}}$. This means that we look for the highest improvement in the MVSK objectives (as measured by *δ*) for which the tilted portfolio is sufficiently close to the initial portfolio. The MVSK portfolio tiling problem is then:

(9) $$\underset{\delta \in R,w\in C}{\text{maximize}}\;\delta \;\text{subject to}{\text{TE}}_{\text{vol}}(w)\leq \tau ,w^{\prime }\mu \geq w_{0}^{\prime }\mu +\delta_{\mu },w^{\prime }\Sigma w\leq w_{0}^{\prime }\Sigma w_{0}-\delta_{\Sigma },w^{\prime }\Phi (w⊗w)\geq w_{0}^{\prime }\Phi (w_{0}⊗w_{0})+\delta_{\Phi },w^{\prime }\Psi (w⊗w⊗w)\leq w_{0}^{\prime }\Psi (w_{0}⊗w_{0}⊗w_{0})-\delta_{\Psi },$$

where ${\left(\delta_{\mu },\delta_{\Sigma },\delta_{\Phi },\delta_{\Psi }\right)}^{\prime }=g(\delta )$ and *τ* determines the maximum tracking error volatility of the tilted portfolio as compared to the reference portfolio.

### 2.3 Shrinkage estimation of comoments

In order to implement the proposed framework, reliable estimates of the comoments ***μ***, **Σ**, **Φ** and **Ψ** are required. One possibility is to use parametric conditions linking the mean estimated return to the estimated comoments, as in Ardia and Boudt (2015). Another possibility is to combine the sample estimator and a parametric structured matrix using the approach of shrinkage estimation, as recommended by Ledoit and Wolf (2003), Martellini and Ziemann (2010) and Boudt et al. (2020a). In general, the shrinkage estimator for the central moments ***θ*** is given by

(10) $$\overset{ˆ}{\theta }=(1-{\overset{ˆ}{\alpha }}^{⋆}){\overset{ˆ}{\theta }}_{\text{sample}}+{\overset{ˆ}{\alpha }}^{⋆}{\overset{ˆ}{\theta }}_{\text{structured}},$$

with ${\overset{ˆ}{\theta }}_{\text{sample}}$ the sample moments and ${\overset{ˆ}{\theta }}_{\text{structured}}$ the moments under a structured estimation approach. Several choices of targets are possible. In this paper, we use the structured moments under the assumption that the marginals are independent, which is a popular choice in practice.

The value of ${\overset{ˆ}{\alpha }}^{⋆}$ is determined in a data-driven way by minimizing the mean squared estimation error:

(11) $$\alpha^{⋆}=\arg\min_{\alpha \in [0,1]}E\left[{\left\|(1-\alpha ){\overset{ˆ}{\theta }}_{\text{sample}}+\alpha {\overset{ˆ}{\theta }}_{\text{structured}}-\theta \right\|}^{2}\right],$$

for which a consistent estimator is derived in Ledoit and Wolf (2003) for the covariance matrix and in Boudt et al. (2020a) for the higher moments.

## 3 Illustration in asset allocation

In this application, we focus on variance-skewness-kurtosis (VSK) efficient portfolios, aiming to decrease the variance and fourth central moment while increasing the third central moment. We do not incorporate the estimated expected returns since their estimation precision is typically low compared to the precision in estimating the portfolio variance, skewness and kurtosis. We further use the following parameters for tilting the portfolio in order to improve the variance, skewness and kurtosis:

(12) $$\text{VSK}:\;\;(\delta_{\mu },\delta_{\Sigma },\delta_{\Phi },\delta_{\Psi })\;=\delta \times (-\infty ,w_{0}^{\prime }\Sigma w_{0},|w_{0}^{\prime }\Phi (w_{0}⊗w_{0})|,w_{0}^{\prime }\Psi (w_{0}⊗w_{0}⊗w_{0})).$$

The VSK tilting above is equivalent to MVSK tilting in case of homogeneous expected asset returns (*i.e.*, $\mu =\mu \iota_{N}$, with $\iota_{N}$ the *N*-dimensional vector of ones and *μ* the common expected return). Note that in (12) we let the function g(δ) depend in a specific way on the benchmark portfolio $w_{0}$. Alternative specifications may be more desirable depending on the application. In particular, when the minimum variance, maximum skewness or minimum kurtosis portfolio is used as benchmark portfolio, the calibration needs to be modified such that a deterioration of the second, third or fourth moment portfolio performance is allowed. This is possible by setting $\delta_{\Sigma }$, $\delta_{\Phi }$, or $\delta_{\Psi }$ to −∞, while setting $h_{\text{ref}}(\cdot )$ to the objective function corresponding to the minimum variance, maximum skewness or minimum kurtosis portfolio.

### 3.1 Data

The data consist of weekly returns of 12 broad scale investment indices over the period January 26, 2001 until March 15, 2019. All series are expressed in EUR. Table 2 provides an overview of the indices along with their mean, volatility, skewness and kurtosis over the full period. The annual volatilities lie between 3% for iBoxx EUR Corporate and 21% for MSCI Emerging Markets. The standardized skewness is generally negative, except for Barclays Global Aggregate Treasuries, US Corporates and World Govt Inflation Linked, for which it is slightly positive. All assets show heavy-tailed behavior and have a positive excess kurtosis.

**Table 2:** Summary statistics of the weekly returns of the asset class indices.

		geom. mean	volatility	skewness	ex. kurt.
1	MSCI Europe	2.82	0.19	-0.71	7.46
2	MSCI USA	4.55	0.18	-0.34	3.05
3	MSCI Japan	1.63	0.19	-0.11	1.31
4	MSCI Emerging Markets	7.35	0.21	-0.15	4.97
5	EPRA Eurozone	5.16	0.19	-1.29	9.01
6	JPM EMU	4.64	0.04	-0.14	2.16
7	iBoxx EUR Corporate	4.50	0.03	-0.94	5.41
8	Bloomberg Barclays Global Aggregate Treasuries	3.17	0.06	0.57	4.01
9	JPM EMBI Global Diversified Composite	7.29	0.11	-0.12	4.87
10	Bloomberg Barclays Global High Yield	6.96	0.10	-0.21	4.70
11	Bloomberg Barclays US Corporates	4.37	0.10	0.29	1.46
12	Bloomberg Barclays World Govt Inflation Linked	4.34	0.07	0.11	1.39

Note: We report the annualized geometric mean (geom. mean, %), the annualized standard deviation (volatility), the standardized skewness and excess kurtosis (ex. kurt.) over the period January 26, 2001 until March 15, 2019.

### 3.2 Illustration of portfolio tilting

**Figure 1:**
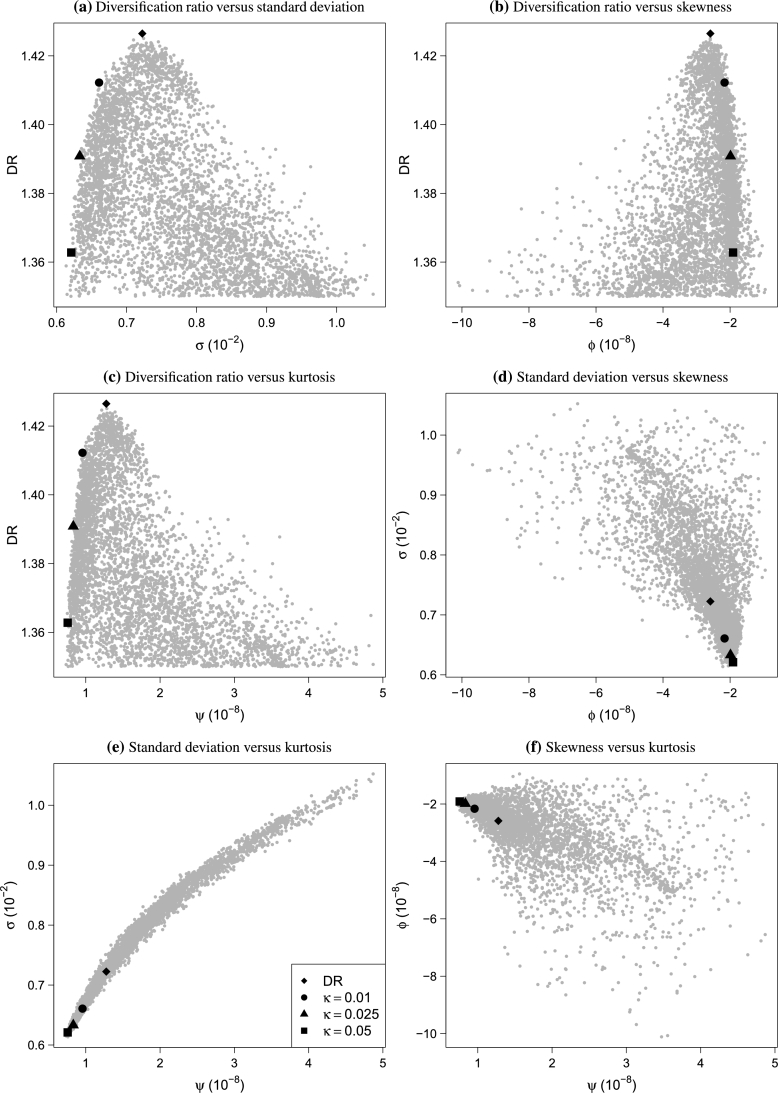
Effect of VSK tilting when the reference portfolio is the maximum diversification portfolio.

**Figure 2:**
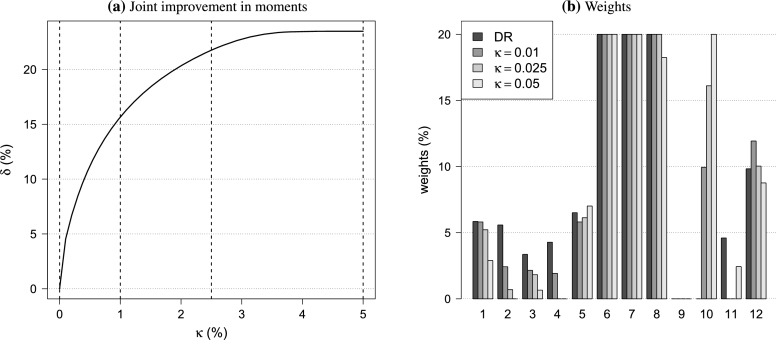
Effect of portfolio tilting on portfolio moments and weights.

Figs. 1 and 2 illustrate the concept of VSK portfolio tilting for the last day of our sample, namely March 15, 2019. The reference portfolio is the maximum diversification portfolio (DR), and the moments are estimated as outlined inbelow.

The dots in Fig. 1 represent different randomly generated feasible portfolios and provide an idea of the set in which we are optimizing. We highlight the DR portfolio and associated tilted portfolios with $\kappa =-(0.01,0.025,0.05)h_{\text{DR}}(w_{0})$. When the margin *κ* increases, the tilted portfolios attain a higher skewness while decreasing in variance and kurtosis.

Fig. 2a shows the joint improvements in the moments measured by the objective value *δ* in (8). It is remarkable how much improvement is possible while only marginally decreasing the diversification ratio. A *κ* of 1% results in a relative improvement of at least 15%, whereas increasing *κ* to 5% yields a relative improvement of around 23%. This is also seen in the bottom row of Fig. 1, where the largest difference between subsequent portfolios occurs when κ=1%.

For the same portfolios, the effect on the weights is illustrated in Fig. 2b. The tilted portfolios lower the equity allocation (assets 1, 2, 3, and 4) and increase the bond component through Global High Yield (asset 10). As mentioned by a referee, bootstrapping can be used to evaluate the estimation uncertainty of the optimized weights or to robustify the weights with respect to estimation uncertainty. We refer the interested reader to Scherer (2002) for a review.

### 3.3 Out-of-sample results

We now turn to analyzing the out-of-sample effects of portfolio tilting on portfolio performance when the reference portfolio is obtained by minimizing $h_{\text{EW}}$, $h_{\text{ERC}}$ and $h_{\text{DR}}$. For $h_{\text{EW}}$ and $h_{\text{ERC}}$, we consider deviations of κ=0.01,0.05,0.10. For $h_{\text{DR}}$, we take *κ* proportional to the diversification ratio of the maximum diversification portfolio; $\kappa =-(0.01,0.025,0.05)h_{\text{DR}}(w_{0})$. In addition to these fixed *κ*, we automatically select the tilted portfolios with maximum annual tracking error volatilities of 0.5% and 1%. We adopt the industry-practice of rolling five-year estimation windows and estimate the moments using the shrinkage approach toward the target that assumes independent marginals. Before estimation, we winsorize the observations following the procedure in Boudt et al. (2008). The portfolios are long-only and fully invested with a maximum weight of 20% in a single asset, complying with UCITS regulations in a fund-of-funds setting. In practice, the indices can be replaced by funds covering different asset classes.

**Figure 3:**
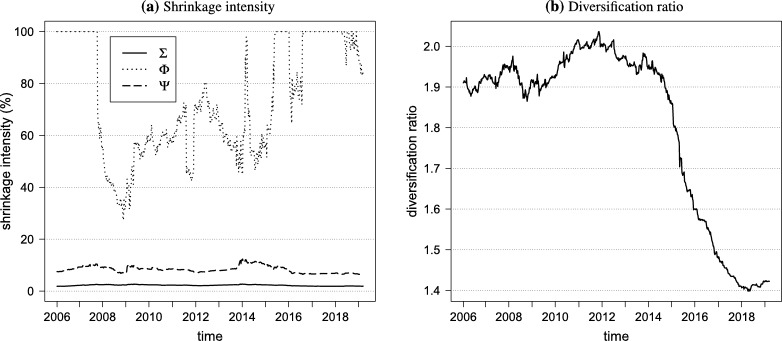
Portfolio statistics over time.

Fig. 3a illustrates that the covariance and cokurtosis shrinkage coefficients are stable over time and put most of the weight on the sample covariance and cokurtosis estimators. The coskewness shrinkage coefficient is more volatile and occasionally puts all weight on the target. This is in line with the findings of Martellini and Ziemann (2010) and Boudt et al. (2020a). The course of the diversification ratio over time is shown in Fig. 3b. Note the large decrease of the diversification ratio in the period 2014 to 2018 due to an increase in the average correlation between the indices from around 24% to 50%.

Table 3 reports summary statistics for the benchmark and tilted portfolios. In all three settings, we observe a decrease in volatility and fourth central moment (*ψ*) and an increase in third central moment (*ϕ*), which is exactly what VSK portfolio tilting aims to achieve. The effect is more pronounced when *κ* increases, but it comes at the cost of a slightly lower average return. As a measure for the trade-off, we follow the recommendation of Zakamouline and Koekebakker (2009) and use the skew-adjusted Sharpe ratio,

(13) $$\text{ASSR}=\text{SR}\sqrt{1+\frac{\text{skewness}}{3}\text{SR}},$$

where the Sharpe ratio (SR) and skewness are computed on the annual out-of-sample returns. The last column of Table 3 shows that tilting improves the ASSR in all cases.

**Table 3:** Portfolio summary: variance-skewness-kurtosis tilting.

		geom. mean	volatility	*ϕ*	*ψ*	MD	Turn.	ASSR
DR		4.506	0.052	-0.222	0.228	-15.605	86.713	0.671
	*κ* = 0.01	4.318	0.049	-0.197	0.190	-13.603	85.650	0.694
	*κ* = 0.025	4.326	0.048	-0.171	0.166	-12.392	92.869	0.718
	*κ* = 0.05	4.473	0.047	-0.147	0.145	-11.053	99.652	0.761
	*τ* = 0.005	4.462	0.049	-0.200	0.195	-13.834	81.661	0.694
	*τ* = 0.01	4.547	0.047	-0.157	0.153	-11.627	90.682	0.755

ERC		4.838	0.053	-0.248	0.264	-14.106	42.527	0.691
	*κ* = 0.01	4.666	0.050	-0.161	0.176	-11.846	63.846	0.738
	*κ* = 0.025	4.611	0.049	-0.130	0.148	-10.795	89.080	0.755
	*κ* = 0.05	4.563	0.048	-0.119	0.134	-10.045	125.653	0.762
	*τ* = 0.005	4.706	0.050	-0.186	0.194	-12.401	82.228	0.711
	*τ* = 0.01	4.688	0.048	-0.144	0.153	-10.768	119.563	0.749

EW		5.447	0.084	-1.305	2.007	-26.562	55.653	0.509
	*κ* = 0.01	5.175	0.066	-0.559	0.713	-19.264	50.015	0.590
	*κ* = 0.025	4.824	0.059	-0.333	0.402	-16.088	95.241	0.617
	*κ* = 0.05	4.670	0.050	-0.197	0.211	-12.570	73.487	0.705
	*τ* = 0.005	5.335	0.079	-1.092	1.566	-25.251	59.247	0.520
	*τ* = 0.01	5.226	0.074	-0.902	1.203	-23.890	68.179	0.532

Note: We report the annualized geometric mean (geom. mean, %), the annualized standard deviation (volatility), the skewness (*ϕ*, $10^{-6}$) and kurtosis (*ψ*, $10^{-7}$), maximum drawdown (%), annual turnover (Turn.) and skew-adjusted Sharpe ratio (ASSR) based on annual returns. The DR, ERC and EW portfolios are reported, along with the performance of their tilted versions for κ=0.01,0.05,0.10 and tilting with a maximum annual tracking error volatility *τ* of 0.5% and 1%.

It is further important to notice in Table 3 that portfolio tilting also improves the downside risk, since the maximum drawdown decreases compared to the benchmark portfolio. In the case of the DR portfolio, the maximum drawdown even goes from -15.6% to -11.1% when allowing a 5% lower diversification ratio. The effect on turnover is highly dependent on the benchmark portfolio. Due to the VSK portfolio being slightly more concentrated, the turnover generally increases with *κ*. However, turnover can be dealt with in practice by imposing an additional constraint in the portfolio optimization step.

**Figure 4:**
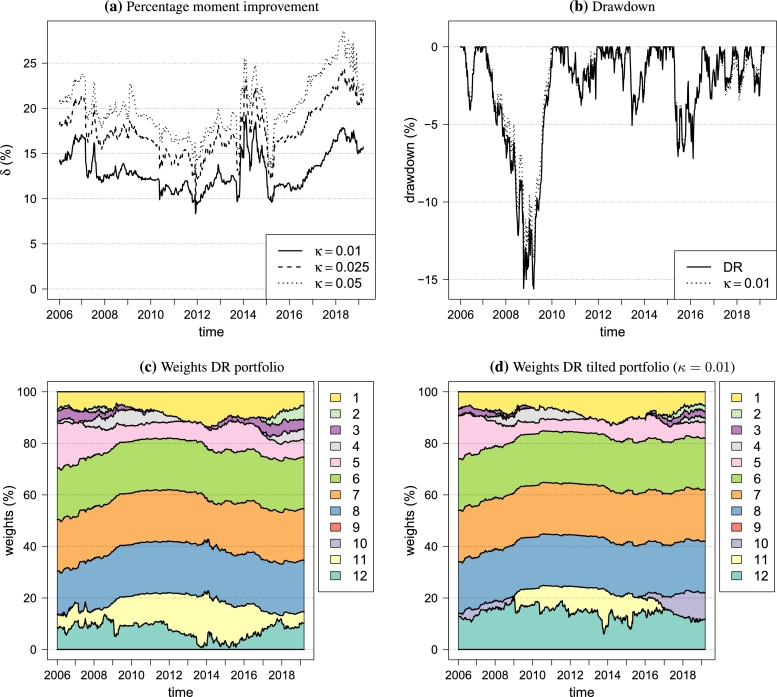
Portfolio statistics over time.

Fig. 4a illustrates the minimum improvement in the variance, skewness and kurtosis as measured by *δ* in (8) when tilting the portfolios. It is clear that most of the improvement happens for low values of *κ*, such as κ=1%. Increasing *κ* is still beneficial, but its effect is dampened. For κ=1%, Fig. 4b shows the difference in drawdown over time between the maximum diversification portfolio and the tilted portfolio. The cause of the difference is found in the weights given in Fig. 4c and 4d. The clearest difference is that the tilted portfolio reduced the overall allocation to equities (assets 1, 2, 3 and 4) while increasing the bond component. Moreover, the tilted portfolio re-allocates part of the bond exposure. The main differences here are the weights of Global High Yield (asset 10) and US Corporates (asset 11). The DR portfolio does not invest in Global High Yield, while the tilted portfolio is invested in the periods 2006-2009 and 2016-2019. Since both are highly correlated and have about the same volatility, this is due to a difference in higher order comoments in relation to the other components of the portfolio.

We verified that the choice of window length, shrinkage target and pre-processing does not alter the conclusion presented in this section. Robustness checks are available from the authors upon request.

## 4 Conclusion

This paper presents a mean-variance-skewness kurtosis (MVSK) portfolio tilting framework designed to modify portfolio weights in a direction that improves the first four moments of the portfolio return distribution. Under the MVSK portfolio tilting framework, the initial portfolio is assumed to be optimal with respect to an initial objective function. However, it is possible to jointly improve the moments at the cost of only a slight deterioration in this initial objective function. Hence, portfolio tilting is a trade-off between the deterioration in portfolio construction objective on the one hand, and the improvements in the portfolio return moments on the other. The empirical application shows the improvements in out-of-sample performance when tilting the equally-weighted, equal-risk-contribution and maximum diversification portfolios in a UCITS-compliant asset allocation setting. These performance gains are low-hanging-fruit for all investors. A practical policy recommendation is therefore that regulators in the field of investor protection should promote knowledge of how higher order moments affect financial portfolio outcomes and how they can be optimized. In order to facilitate this process, we have released an open source implementation in the R package **mvskPortfolios**.

## Declarations

### Author contribution statement

K. Boudt, D. Cornilly, F. Van Holle and J. Willems: Conceived and designed the experiments; Performed the experiments; Analyzed and interpreted the data; Contributed reagents, materials, analysis tools or data; Wrote the paper.

### Funding statement

The authors gratefully acknowledge support from the Research Foundation - Flanders (FWO research grant G023815N and PhD fellowship 1114117*N*).

### Competing interest statement

The authors declare no conflict of interest.

### Additional information

The analysis can be reproduced using the code available at https://github.com/cdries/mvskPortfolios.

Supplementary content related to this article has been published online at https://doi.org/10.1016/j.heliyon.2020.e03516.

No additional information is available for this paper.

## Supplementary material

The following Supplementary material is associated with this article:

Appendix.pdf In this supplementary appendix to the paper Boudt et al. (2020), we provide a brief R tutorial for the proposed MVSK portfolio tilting. Appendix.pdf

## Footnotes

1 This approach of optimizing non-standardized moments is consistent with the results of Scott and Horvath (1980) which support preferences for third and fourth central moments instead of the standardized skewness and excess kurtosis. We use tilting in the sense of over- or underweighting assets compared to benchmark portfolios (see e.g. Darsinos and Satchell (2004)). We refer the reader to Stutzer, 2000, Stutzer, 2003, Haley and McGee (2006), Haley and Whiteman (2008) and Haley and McGee (2011) for an alternative definition of tilting in a portfolio context, namely modifying the probability return distribution.
