# Supplementary material for: Algorithmic portfolio tilting to harvest higher moment gains
Source: Heliyon. 2020 Mar 5;6(3):e03516. doi: 10.1016/j.heliyon.2020.e03516 (PMC7062767; doi:10.1016/j.heliyon.2020.e03516)
Supplement: Appendix.pdf — In this supplementary appendix to the paper Boudt et al. (2020), we provide a brief R tutorial for the proposed MVSK portfolio tilting. [file mmc1.pdf]

Supplementary appendix to:
Algorithmic portfolio tilting to harvest higher moment gains

Kris Boudt
Kris Boudt is professor of finance and econometrics at Universiteit Gent, Vrije Universiteit Brussel and Vrije Universiteit Amsterdam
Address: Sint-Pietersplein 5, 9000 Gent, Belgium
Email: kris.boudt@ugent.be

Dries Cornilly
Dries Cornilly is quantitative analyst at SYZ Asset Management
Address: Rue du Commerce 3. 1204 Genève, Switzerland
Email: dries.cornilly@syzgroup.com

Frederiek Van Holle
Frederiek Van Holle is head of Quant Solutions at Degroof Petercam Asset Management
Address: Guimardstraat 18, 1040 Brussel, Belgium
Email: f.vanholle@degroofpetercam.com

Joeri Willems
Joeri Willems is a quantitative fund manager at Degroof Petercam Asset Management
Address: Guimardstraat 18, 1040 Brussel, Belgium
Email: j.willems@degroofpetercam.com

Abstract

In this supplementary appendix to the paper Boudt et al. (2020), we provide a brief `R` tutorial for the proposed MVSK portfolio tilting.
Keywords: Mean-variance-skewness-kurtosis, non-normality, portfolio allocation, tilting.
JEL codes: C100, G110

---

1. Implementation in the R package mvskPortfolios

The empirical application is performed using the R package **mvskPortfolios**. The developer version can be installed from GitHub by

```
library(devtools)
install_github("cdries/mvskPortfolios")
```

The main function to construct tilted portfolios is **mvskPortfolio**. The principal arguments are the expected returns (`m1`), covariance matrix (`M2`), coskewness matrix (`M3`) and cokurtosis matrix (`M4`). The parameter `g` is either equal to the vector $(\delta_\mu, \delta_\Sigma, \delta_\Phi, \delta_\Psi)$ or a custom function $g(\delta)$. The default MVSK choice is accessible by `g="mvsk"` and is equivalent to VSK, used in the paper, when `m1` is not supplied to the **mvskPortfolio** function. The options for `w0` include the optimized portfolios (`DR`, `ERC`, `EW`) as well as the possibility of passing a vector with custom weights. The parameter (vector) `kappa` determines the tolerance on the objective specified in `href`. Below, we impose a maximum allocation of 30% to a single asset through the argument `ub`.

As an example, we load the edhec data from the **PerformanceAnalytics** package and use shrinkage estimation to estimate the covariance matrix (`M2`), coskewness matrix (`M3`) and cokurtosis matrix (`M4`):

```
library(PerformanceAnalytics)
# load edhec hedge fund style return data
data(edhec)
x <- edhec[, 1:5] * 100
# estimate comoment matrices
M2 <- M2.shrink(x, targets = 1)$M2sh
M3 <- M3.shrink(x, targets = 1)$M3sh
M4 <- M4.shrink(x, targets = 1)$M4sh
```

We then compute the VSK portfolio with the maximum diversification portfolio as reference and with other parameters set to the defaults used in this paper:

```
library(mvskPortfolios)
resVSK <- mvskPortfolio(M2 = M2, M3 = M3, M4 = M4, w0 = "DR",
                g = "mvsk", ub = rep(0.3, 5), href = "DR",
                kappa = c(0, 0.01, 0.025, 0.05), relative = TRUE)
```

The output then includes the weights for each of the values in `kappa`, as well as the corresponding objective values δ. In addition, the portfolio moments and inequality constraints are returned. The latter step is taken to allow the user to easily check which constraints are binding. Detailed documentation of the function is available by running `?mvskPortfolio`.

References

Boudt, K., Cornilly, D., Van Holle, F., and Willems, J. (2020). Algorithmic portfolio tilting to harvest higher moment gains. *Heliyon*.
